# Reduce the risk of microbial activity and cytotoxicity by *Adansonia digitata* pulp extract grown under the semi arid conditions of Sudan

**DOI:** 10.1038/s41598-025-30536-x

**Published:** 2025-12-02

**Authors:** Rasha Khalid Abbas

**Affiliations:** Department of Chemistry, Faculty of Science, AL-Baha University Saudi Arabia, P.O. Box 65931, AL-Baha city, Saudi Arabia

**Keywords:** Adansonia digitata, HPLC, *Bacillus subtilis* (ATCC 6633), *Escherichia coli* (ATCC 8739), *Salmonella Typhi* (ATCC 6539), Fungi *Candida albicans* (ATCC 10221), *Aspergillus Niger* ATCC 16888), (MIC), MBC, Antibiotics gentamicin, Fluconazole: cancer cell line hela, Hep G2, A549, A-431, Pc3, T-47D, Biotechnology, Cancer, Drug discovery, Microbiology, Plant sciences

## Abstract

Sudan is rich country for natural products like *Adansonia digitata* is locally name Gongolase and Tabldy, this plant has gained an interest for study because have many therapeutic benefits and low side effects and have high demand in the world market and is expected to be replace chemical products. Plant pulp extracts contain chemical constituents such as polyphenol, which are responsible for Antimicrobial activity and cytotoxic activity. The current studied investigates of the edible parts of *Adansonia digitata* ethanoic pulp extract against bacteria, fungi and anticancer. HPLC used to identify the polyphenol compound, agar well diffusion method used to estimate the antimicrobial activity, the method of dilution in liquid medium was used for the determination of the minimum inhibitory concentration (MIC), (MBC) and to estimate anticancer of pulp edible parts of *Adansonia digitata* used MTT protocol. Polyphenol constituents of *Adansonia digitata* pulp extracts was examined by HPLC contained compounds have antimicrobial activity to pathogenic bacteria (*Bacillus subtilis* (ATCC 6633), *Escherichia coli* (ATCC 8739), *Salmonella typhi* (ATCC 6539), fungi *Candida albicans* (ATCC 10221), *Aspergillus niger* ATCC 16888) compare with antibiotic for bacteria (Gentamicin) and for fungi (Fluconazole), also have cytotoxicity against six cancer cell line such as, Hela (The line is derived from cervical cancer), Hep G2 (a human liver cancer), A549 (lung cancer), A-431 (cells epidermis carcinoma), PC3 (a human prostate cancer) and T-47D (human breast cancer cell line).

**Supplementary Information:**

The online version contains supplementary material available at 10.1038/s41598-025-30536-x.

## Introduction

 The secondary metabolites like phenolic compounds, alkaloids, sulphur-containing compounds and terpenoids of plant parts are effective against the disease-causing agents, which have high efficacy and lower toxicity as compared to synthetic drugs^1–3^. Since they are presently recognized as therapeutically useful molecules, which can be potential alternatives for the development of anticancer drugs and antimicrobial^4,5^.The variability in agro-climatic conditions in Sudan is characterized it wide range of different crops can be grown throughout the year, one of these crops is *Adansonia digitata*. Locally names “ Gongolase, Tabldy, Baobab, monkey-bread tree, the dead-rat tree (owing to its fruit shape), is a large iconic tree, majestic sub-tropical tree belongs to the family Malvaceae, known multipurpose tree, that grows and originates in the sub-Sahara areas of Africa it found in Sudan, Nigeria, Mali, Burkina Faso, Senegal, Kenya^6–13^. All different parts of Adansonia tree (roots, trunk, bark, leaves, pulp and seeds) beneficial to human used for therapeutic purposes such as anticancer, antimicrobial, antiviral and anti-trypanosome activity, against stomachaches and diarrhea^14–21^. *Adansonia digitata* tree Have high nutritional value and unique flavor Baobab leaves are good source of protein with essential amino acids vitamins (such as vitamin A, vitamin C, and vitamin B6), minerals (such as calcium, iron, and potassium), dietary fibre, and as well as palmitic, oleic, and linoleic acids^21–30^. They also contain beneficial bioactive compounds, including polyphenols and flavonoids, which contribute to their potential health benefits. Baobab leaves used in the preparation of sauces, soups, stews, and other traditional dishes in Africa. They impart a tangy and slightly sour taste to the dishes, adding a unique flavor furthermore; baobab pulp extracts have antimicrobial and antioxidant^30–35^. Medicinal plants can be an interesting source of new antibiotic compounds, which could possibly help tacking the problem of resistance to antibiotics, also used as anticancer. *Adansonia Digitata* often used in the traditional treatment of various infectious diseases, anticancer and have high nutritive value especially in Africa (especially in Sudan).

The objective of this study:-

To identify the polyphenol of *Adansonia digitata* pulp by HPLC that have activity against microorganism and cancer cells.

## Materials and methods

### Plant material

**Fig. 1:**
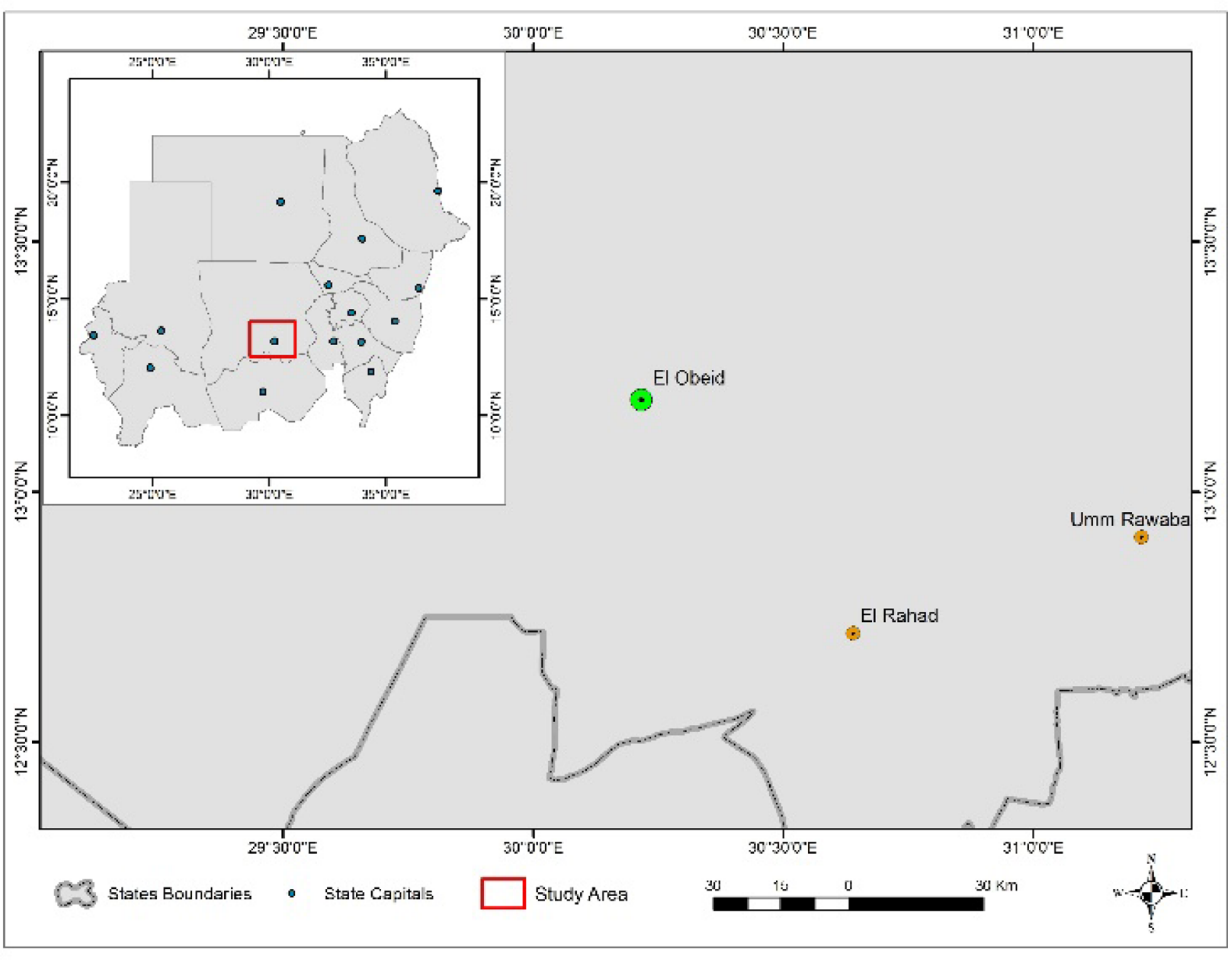
EI-Obeid (north Kordofan State) west Sudan.

(Supplementary Material) Mature fruits pulp of *Adansonia digitata* specimen 436/2022, deposited at MAPRI herbarium, National Center for Research, Sudan, identified by Dr. Mubark Sidig was purchased from Omer Bin Khatab market (previously Abu Jahl market) at EI-Obeid (north Kordofan State) west Sudan, Fig. 1.

### Extraction and Preparation of extracts

The powder separated from seeds manually and crushed using mortar and pestle to coarse powder then macerated with 80%ethanol at room temperature for 24 h, filtered and evaporated using rotary evaporator and kept in a dry brown container at refrigerator until used.

High Performance Liquid Chromatography device.

### Microorganisms

In this study, a conventional biochemical method used to identify the microorganism in the three separate laboratories according to standard microbiology techniques. The pathogenic were, *Bacillus subtilis* (ATCC 6633), *Escherichia coli* (ATCC 8739) and *Salmonella typhi* (ATCC 6539, fungus *Candida albicans* (ATCC 10221) and *Aspergillus niger* (ATCC 16888).

## Methods

### Agar well diffusion method

In this method used Nutrient agar for antibacterial activity and potato dextrose agar for antifungal activity, Agar well diffusion method is widely used to evaluate the antimicrobial activity of plants or microbial extracts, the agar plate surface was inoculated by spreading a volume of the microbial inoculum over the entire agar surface. Then, a hole with a diameter of 6 to 8 mm punched aseptically with a sterile cork borer or a tip, and a volume (20–100mL) of the antimicrobial agent or extract solution at desired concentration. Then, agar plates incubated under suitable conditions depending upon the test microorganism. the antimicrobial agent diffuses in the agar medium and inhibits the growth of the microbial strain tested^35–38^.

### Determination of minimum inhibitory concentration (MIC) and minimum bactericidal concentration (MBC)

The method of dilution in liquid medium was used for the determination of the minimum inhibitory concentration (MIC), all the inoculated dilutions are incubated for 24 h at 37 C, and the results are read according to the turbidity. The nutrient agar poured into petri dishes is inoculated in streaks with 100 µl of the contents of the tubes having a concentration ≥ MIC in the previous dilution series. Minimum bactericidal concentration (MBC) is determined after incubation for 24 h at 37 C. It is the smallest concentration that completely inhibits growth. In addition, the MBC/MIC ratio of each extract is calculated in order to assess its antibacterial power. When the MBC/MIC ratio of a given substance is less than or equal to 4, this substance is considered bactericidal, if this ratio is greater than 4 it is said to be bacteriostatic^39,40^.

### Determination of sample cytotoxicity on cells (MTT protocol)

To develop a complete monolayer sheet inoculated the 96 well tissue culture plate with 1 × 10^5^ cells/ml (100 ul/well) and incubated at 37 °C for 24 h. Growth medium was decanted from 96 well microliter plates after a confluent sheet of cells were formed, cell monolayer was washed twice. Two-fold dilutions of the tested sample were made in RPMI medium with 2% serum 0.1 ml of each dilution was tested in different wells leaving three wells as control; Plate was incubated at 37 °C and examined. Cells checked for any physical signs of toxicity, e.g. partial or complete loss of the monolayer, rounding, shrinkage, or cell granulation. MTT solution was prepared (5 mg/ml in PBS) (BIO BASIC CANADA INC). 20ul MTT solution added to each well. Place on a shaking table, 150 rpm for 5 minutes, to mix thoroughly the MTT into the media. Incubate (37 C, 5% CO2) for 4 h to allow the MTT to be metabolized. Dump off the media. Suspend Formosan (MTT metabolic product) in 200ul DMSO. Place on a shaking table, 150 rpm for 5 minutes, to mix thoroughly the Formosan into the solvent. Read optical density at 560 nm and subtract background at 620 nm. Optical density should be directly correlated with cell quantity^39^.

### Statistical analysis

Minitab version 17 software used to analyze all data. The mean and Standard deviation were calculated. Regression analysis used to evaluate the significant difference. A linear regression equation was used to determine the half-maximal inhibitory concentration IC50 from the linear part of the sigmoid curve in order to analyze cell viability, i.e. y = mx + c, y = 50, M and C values were derived from the viability graph. R 2 values ≥ 0.95 were considered statistically significant.

### HPLC conditions

(Supplementary Material) Using an Agilent 1260 series for HPLC analysis. Using Zorbax Eclipse plus C8 column (4.6 mm x 250 mm i.d., 5 μm) for the separation. The mobile phase consisted of water (A) and 0.05% trifluoroacetic acid in acetonitrile (B) at a flow rate 0.9 ml/min. The programmed mobile phase was consecutively in a linear gradient as follows: 0 min (82% A); 0–1 min (82% A); 1–11 min (75% A); 11–18 min (60% A); 18–22 min (82% A); 22–24 min (82% A). The multi-wavelength detector monitored at 280 nm. The volume for injection was five µl for each of the sample solutions. The temperature of the column at 40 °C.

## Results and discussion

**Fig. 2:**
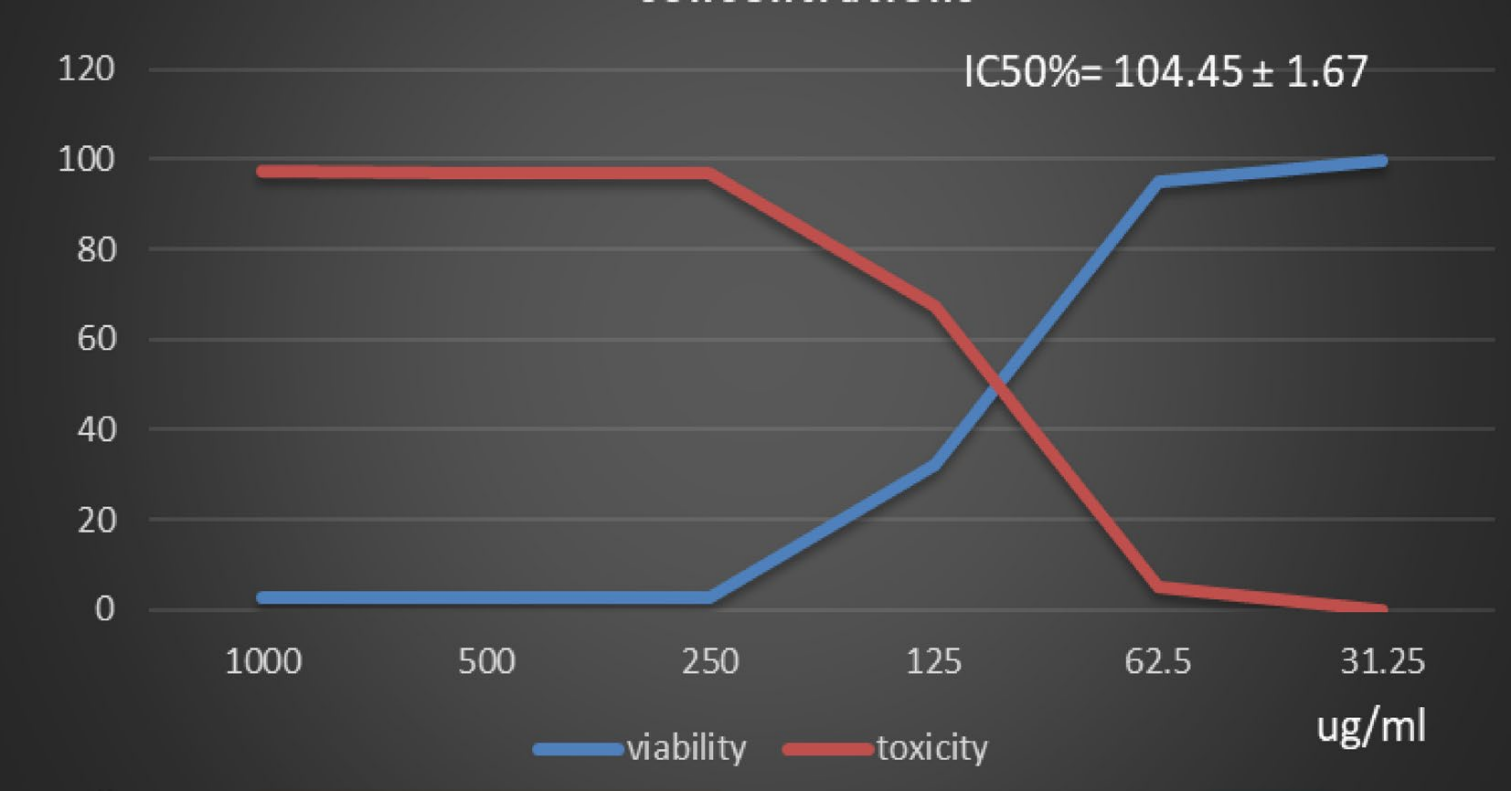
Effect of *Adansonia digitata* pulp extracts against Hela cell at different concentration.

**Fig. 3:**
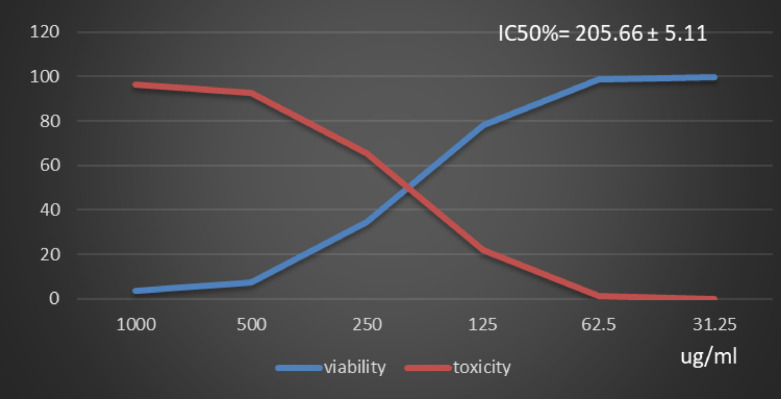
Effect of *Adansonia digitata* pulp extracts against HepG2 cell at different concentration.

**Fig. 4:**
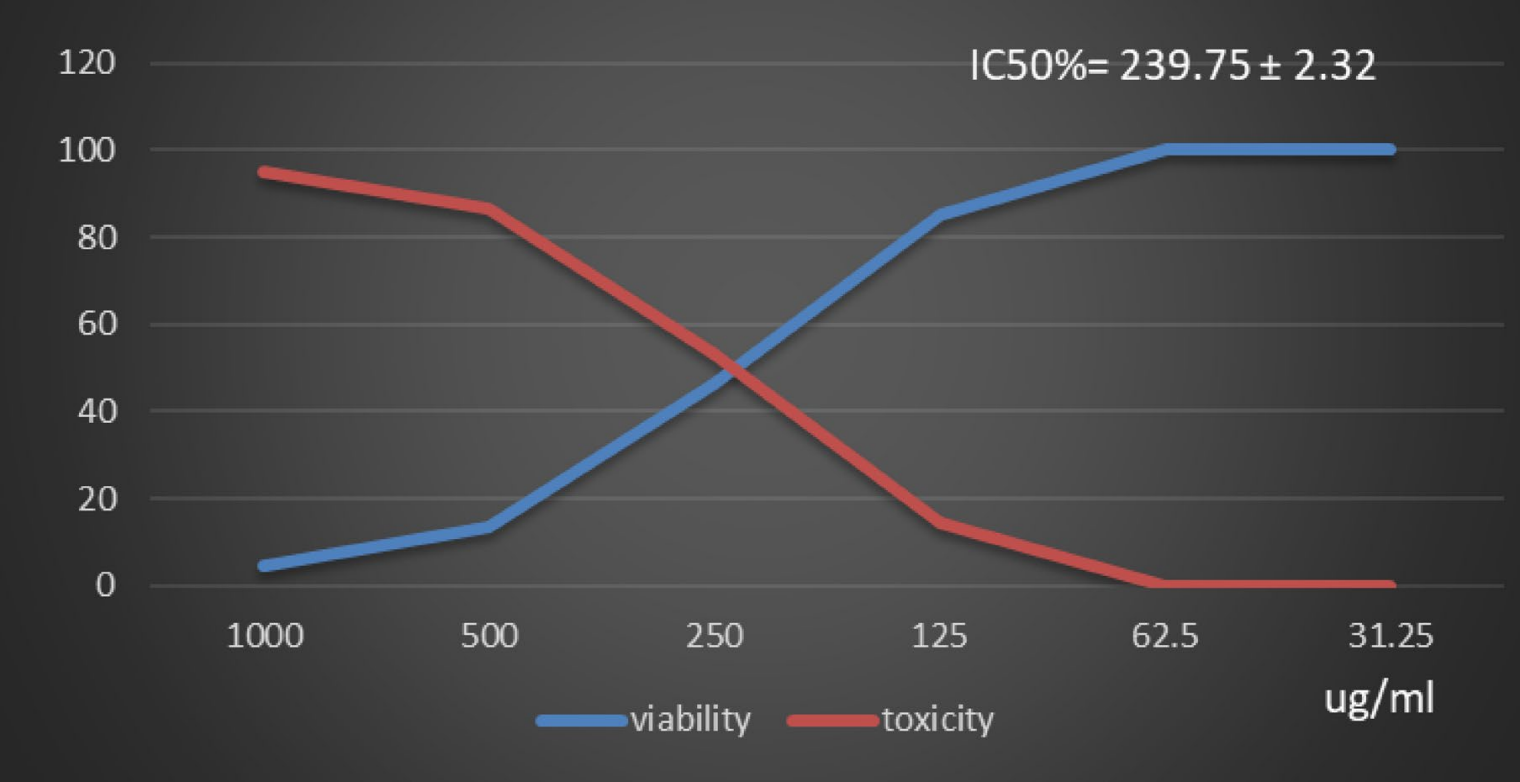
Effect of *Adansonia digitata* pulp extracts against A549 cell at different concentration.

**Fig. 5:**
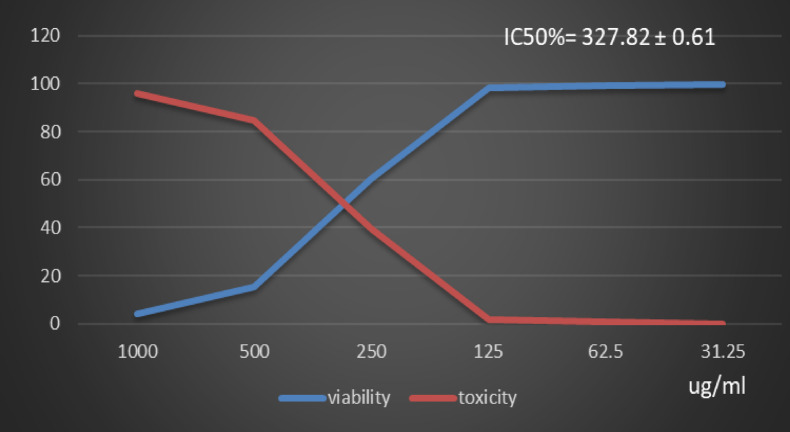
Effect of *Adansonia digitata* pulp extracts against A431 cell at different concentration.

**Fig. 6:**
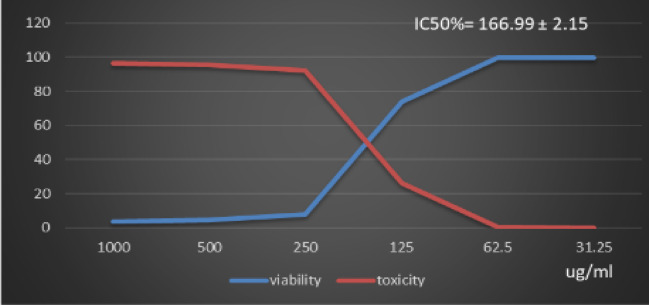
Effect of *Adansonia digitata* pulp extracts against Pc3 cell at different concentration.

**Fig. 7:**
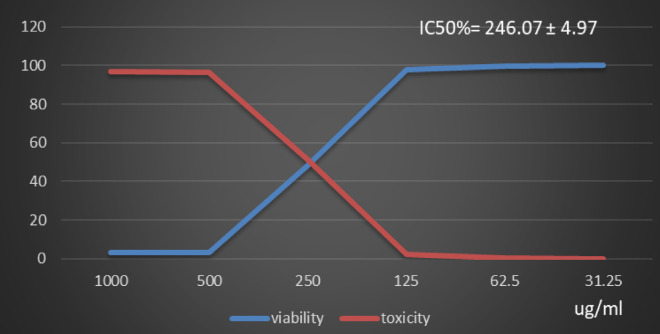
Effect of *Adansonia digitata* pulp extracts against T47D cell at different concentration.

Table 1 and (Supplementary Material). Showed the Polyphenol constituents of *Adansonia digitata* pulp extract contained 18 compounds, 4 of them were major compounds Ellagic acid (8.3624%), Coumaric acid (8.8411%), Rosmarinic acid (7.3747%) and Cinnamic acid (8.5964%) these results agree with those who said that the primary bioactive constituents found in *Adansonia digitata* have antibacterial, anti-inflammatory, antioxidant, anticancer, cardio protective, neuroprotective, and antidiabetic activity^31,38^. Table 2 Showed that moderate inhibitions zones of *Adansonia digitata* ethanoic pulp extracts against pathogenic bacteria (*Bacillus subtilis* (ATCC 6633), *Escherichia coli* (ATCC 8739) and *Salmonella typhi* (ATCC 6539) with an inhibition diameter of 25 ± 0.1 mm, 23 ± 0.1 mm and 26 ± 0.2 mm respectively, compare with antibiotic Gentamicin inhibition zones diameter to above pathogenic bacteria were 28 ± 0.2 mm, 32 ± 0.1 mm and 29 ± 0.2 mm respectively, as well as fungi *(Candida albicans* (ATCC 10221) and *Aspergillus niger* (ATCC 16888)) 21 ± 0.2 mm and 11 ± 0.1 mm respectively compare with antibiotic fluconazole, inhibition diameter 26 ± 0.2 mm and 27 ± 0.1 mm respectively, Table 3 showed minimum inhibitory concentration (MIC) (g/ml), minimum bactericidal concentration (MBC) (g/ml) and MBC/MIC values The ethanoic extract of the pulp of Adansonia Digitata showed a bactericidal effect on the all bacteria and fungi tested, this due to present of polyphenol (Ellagic acid, Coumaric acid, Rosmarinic acid and Cinnamic acid), Ellagic acid can damage bacterial cell walls and membranes, while coumaric, rosmarinic, and cinnamic acids primarily act by binding to and inactivating proteins and enzymes, disrupting membrane function, and inducing oxidative stress. (Supplementary Material) Showed inhibitions zones (in mm) by Adansonia *digitata* ethanoic pulp extracts against pathogenic bacteria (*Bacillus subtilis* (ATCC 6633), *Escherichia coli* (ATCC 8739), *Salmonella typhi* (ATCC 6539) respectively. (Supplementary Material) showed the inhibitions zones (in mm) by *Adansonia digitata* ethanoic pulp extracts against fungi *Candida albicans (ATCC 10221)* and *Aspergillus niger* (ATCC 16888) respectively, these finding are in agreement with that previously studies^2,12^. Table 4, in addition Fig. 2 Showed the cytotoxicity of *Adansonia digitata* pulp extracts against Hela cell line (cervical cancer) at different concentrations, the IC_50_ (50% growth inhibition) was 104.45 ± 1.67, (Supplementary Material) Hela cell Control. (Supplementary Material) Showed the effect of *Adansonia digitata* pulp extracts against Hela cell at different concentration (1000, 500, 250,125, 62.5 and 31.25 ug/ml), showed an excellent cytotoxicity at high concentration 1000,500 and 250 ug/ml this due to widely distributed polyphenols in plants that possess anti-inflammatory and anticancer these finding are in agreement with that previously studies^39–43^. Table 5; Fig. 3 Showed the cytotoxicity of the *Adansonia digitata* pulp extract against HepG2 cell line (a human liver cancer) at different concentrations found the IC_50_ (50% growth inhibition) was 205.66 ± 5.11(Supplementary Material) HepG2 cell Control. (Supplementary Material) Showed the effect of *Adansonia digitata* pulp extracts against HepG2 cell at different concentration (1000, 500, 250,125, 62.5 and 31.25 ug/ml), found cytotoxicity at high concentration 1000 and 500 ug/ml that main the plant contain beneficial bioactive compounds, including polyphenols and flavonoids, which contribute to their potential health benefits these results agree with those studies^44–47^. Table 6. In addition, Fig. 4 found that the cytotoxicity of the *Adansonia digitata* pulp extract were conducted using A549 cell line (lung cancer) at different concentrations found the IC_50_ (50% growth inhibition) was 239.75 ± 2.32. (Supplementary Material) A549 cell Control. (Supplementary Material) Showed the effect of *Adansonia digitata* pulp extracts against A549 cell at different concentration (1000, 500, 250,125, 62.5 and 31.25 ug/ml), showed cytotoxicity at high concentration 1000 and 500 ug/ml these finding are in agreement with that previously reported studies^46,47^.Table 7; Fig. 5 showed the cytotoxicity of the pulp of *Adansonia digitata* were conducted using A431 cell line (cells epidermis carcinoma) at different concentrations, the IC_50_ (50% growth inhibition) was 327.82 ± 0.61. (Supplementary Material) A431 cell Control. (Supplementary Material) Showed the effect of *Adansonia digitata* pulp extracts against A431 cell at different concentration (1000, 500, 250,125, 62.5 and 31.25 ug/ml), showed cytotoxicity at high concentration 1000and 500 ug/ml these finding are in agreement with that previously reported studies^46,47^.Table 8; Fig. 6. Showed that the cytotoxicity of the pulp of *Adansonia digitata* were conducted using Pc3 cell line (prostate cancer) at different concentrations, the IC_50_ (50% growth inhibition) was166.99 ± 2.15. (Supplementary Material) Pc3 cell Control. (Supplementary Material) Showed the effect of *Adansonia digitata* pulp extracts against Pc3 cell at different concentration (1000, 500, 250,125, 62.5 and 31.25 ug/ml), showed cytotoxicity at high concentration 1000,500 and 250 ug/ml these finding are in agreement with that previously reported studies^47^.Table 9; Fig. 7 Showed the cytotoxicity of *Adansonia digitata* pulp were conducted using T47D cell line (human breast cancer) at different concentrations, the IC_50_ (50% growth inhibition) was 246.07 ± 4.97, (Supplementary Material) T47D cell Control. (Supplementary Material) Showed the effect of *Adansonia digitata* pulp extracts against T47D cell at different concentration (1000, 500, 250,125, 62.5 and 31.25 ug/ml), the pulp of *Adansonia digitata* showed cytotoxicity at high concentration 1000 and 500 ug/ml, baobab pulp extract rich content of bioactive compounds like polyphenols and flavonoids so that exerts cytotoxic effects on cancer cells by inhibiting cell proliferation and inducing apoptosis, while the exact mechanisms are still under investigation, baobab compounds can interfere with cellular pathways like the PI3K/Akt signaling cascade, a key regulator of cell growth and survival, leading to increased programmed cell death and reduced cell proliferation, with further research needed to fully elucidate the specific molecular pathways involved in cancer types, these finding are in agreement with that previously reported studies^45–49^.

**Table 1:** Polyphenol constituents of *Adansonia digitata* pulp extract identified by HPLC.

Name of the Poly phenol compound	Ret. Time	Area%
Gallic acid	3.572	3.6540
Chlorogenic acid	4.183	5.7638
Catechin	4.423	4.8432
Methyl gallate	5.413	4.2906
Coffeic acid	5.823	3.2464
Syringic acid	6.313	4.2625
Rutin	6.722	6.0454
Ellagic acid	7.102	8.3624
Coumaric acid	8.542	8.8411
Vanillin	8.967	5.1506
Ferulic acid	9.574	5.2579
Naringenin	10.194	4.9199
Rosmarinic acid	11.610	7.3747
Daidzein	15.792	4.7034
Querectin	17.151	4.4204
Cinnamic acid	19.112	8.5964
Kaempferol	20.502	4.1067
Hesperetin	21.095	6.1607

**Table 2:** Inhibition zone (in mm) by *Adansonia digitata* pulp extract.

Pathogenic microorganism	Inhibition zone (in mm)	Control
*Bacillus subtilis (ATCC 6633)*	25 ± 0.1	28 ± 0.2
*Escherichia coli (ATCC 8739)*	23 ± 0.1	32 ± 0.1
*Salmonella typhi (ATCC 6539)*	26 ± 0.2	29 ± 0.2
*Candida albicans (ATCC 10221)*	21 ± 0.2	26 ± 0.2
*Aspergillus niger* ATCC 16,888	11 ± 0.1	27 ± 0.1

* NA : No activity.

* Control for Bacteria was Gentamicin and for fungi was Fluconazole.

**Table 3:** Minimum inhibitory concentration (MIC) (g/ml). Minimum bactericidal/fungcidal concentration (MBC) (g/ml) and MBC/MIC values by *Adansonia digitata* pulp extract.

Bacteria	MIC	MBC	MBC/MIC
Bacillus subtilis (ATCC 6633)	0.09	0.19	2.1
Escherichia coli (ATCC 8739)	0.17	0.33	1.94
Salmonella typhi (ATCC 6539)	0.17	0.31	1.82

Fungi	MIC	MFC	MFC/MIC
Candida albicans (ATCC 10221)	0.06	0.16	2.7
Aspergillus niger ATCC 16,888	0.13	0.46	3.5

**Table 4:** Effect of *Adansonia digitata* pulp extracts against Hela cell at different concentration.

ID	ug/ml	O.D			Mean O.D	±SE	Viability %	Toxicity %	IC50 ± SD
Hela	-	0.663	0.658	0.656	0.659	0.002082	100	0.739	ug
*Adansonia digitata* pulp extracts	1000	0.018	0.017	0.02	0.018333	0.000882	2.781992919	97.21800708	104.45 ± 1.67
	500	0.022	0.018	0.019	0.019667	0.001202	2.984319676	97.01568032	
	250	0.019	0.019	0.02	0.019333	0.000333	2.933737987	97.06626201	
	125	0.22	0.197	0.224	0.213667	0.008413	32.42286292	67.57713708	
	62.5	0.626	0.619	0.631	0.625333	0.00348	94.89124937	5.108750632	
	31.25	0.658	0.657	0.658	0.657667	0.000333	99.79767324	0.202326758	

**Table 5:** Effect of *Adansonia digitata* pulp extracts against HepG2 cell at different concentration.

ID	ug/ml	O.D			Mean O.D	±SE	Viability %	Toxicity %	IC50 ± SD
HepG2	-	0.747	0.749	0.745	0.747	0.001155	100	0.739	ug
*Adansonia digitata* pulp extracts	1000	0.024	0.033	0.028	0.028333	0.002603	3.792949576	96.20705042	205.66 ± 5.11
	500	0.074	0.052	0.044	0.056667	0.008969	7.585899152	92.41410085	
	250	0.24	0.274	0.263	0.259	0.010017	34.67202142	65.32797858	
	125	0.563	0.598	0.586	0.582333	0.010269	77.95626952	22.04373048	
	62.5	0.736	0.741	0.735	0.737333	0.001856	98.70593485	1.294065149	
	31.25	0.745	0.748	0.744	0.745667	0.001202	99.82150826	0.178491745	

**Table 6:** Effect of *Adansonia digitata* pulp extracts against A549 cell at different concentration.

ID	ug/ml	O.D			Mean O.D	±SE	Viability %	Toxicity %	IC50 ± SD
A549	-	0.65	0.646	0.642	0.646	0.002309	100	0.739	Ug
*Adansonia digitata* pulp extracts	1000	0.034	0.031	0.028	0.031	0.001732	4.79876161	95.20123839	239.75 ± 2.32
	500	0.084	0.099	0.075	0.086	0.007	13.3126935	86.6873065	
	250	0.3	0.294	0.307	0.300333	0.003756	46.49122807	53.50877193	
	125	0.56	0.556	0.537	0.551	0.007095	85.29411765	14.70588235	
	62.5	0.651	0.643	0.644	0.646	0.002517	100	0	
	31.25	0.649	0.644	0.645	0.646	0.001528	100	0	

**Table 7:** Effect of *Adansonia digitata* pulp extracts against A431 cell at different concentration.

ID	ug/ml	O.D			Mean O.D	±SE	Viability %	Toxicity %	IC50 ± SD
A431	-	0.734	0.731	0.737	0.734	0.001732	100	0.739	Ug
*Adansonia digitata* pulp extracts	1000	0.034	0.03	0.028	0.030667	0.001764	4.178019982	95.82198002	327.82 ± 0.61
	500	0.116	0.103	0.118	0.112333	0.004702	15.30426885	84.69573115	
	250	0.438	0.45	0.441	0.443	0.003606	60.35422343	39.64577657	
	125	0.72	0.716	0.725	0.720333	0.002603	98.13805631	1.861943688	
	62.5	0.733	0.727	0.725	0.728333	0.002404	99.22797457	0.772025431	

**Table 8:** Effect of *Adansonia digitata* pulp extracts against Pc3 cell at different concentration.

ID	ug/ml	O.D			Mean O.D	±SE	Viability %	Toxicity %	IC50 ± SD
Pc3	-	0.549	0.55	0.545	0.548	0.001528	100	0.739	Ug
*Adansonia digitata* pulp extracts	1000	0.019	0.02	0.02	0.019667	0.000333	3.588807786	96.41119221	166.99 ± 2.15
	500	0.024	0.023	0.026	0.024333	0.000882	4.440389294	95.55961071	
	250	0.045	0.038	0.049	0.044	0.003215	8.02919708	91.97080292	
	125	0.418	0.396	0.402	0.405333	0.006566	73.96593674	26.03406326	
	62.5	0.545	0.538	0.552	0.545	0.004041	99.45255474	0.547445255	
	31.25	0.549	0.551	0.542	0.547333	0.002728	99.8783455	0.121654501	

**Table 9:** Effect of *Adansonia digitata* pulp extracts against T47D cell at different concentration.

ID	ug/ml	O.D			Mean O.D	±SE	Viability %	Toxicity %	IC50 ± SD
T47D	-	0.765	0.763	0.767	0.765	0.001155	100	0.739	Ug
*Adansonia digitata* pulp extracts	1000	0.021	0.029	0.025	0.025	0.002309	3.267973856	96.73202614	246.07 ± 4.97
	500	0.024	0.028	0.027	0.026333	0.001202	3.442265795	96.5577342	
	250	0.358	0.385	0.366	0.369667	0.008007	48.32244009	51.67755991	
	125	0.752	0.739	0.748	0.746333	0.003844	97.55991285	2.440087146	
	62.5	0.766	0.758	0.762	0.762	0.002309	99.60784314	0.392156863	
	31.25	0.763	0.769	0.763	0.765	0.002	100	0	

## Conclusion

This work comes to conclude that the *Adansonia digitation* pulp extracts contain polyphenol compounds have activity against pathogenic microorganism *Bacillus subtilis* (ATCC 6633), *Escherichia coli* (ATCC 8739), *Salmonella typhi* (ATCC 6539), fungi *Candida albicans* (ATCC 10221), *Aspergillus niger* ATCC 16888) compare with antibiotic for bacteria (Gentamicin) and for fungi (Fluconazole), also have cytotoxicity against six cancer cell line such as Hela (The line is derived from cervical cancer), Hep G2 (a human liver cancer), A549 (lung cancer), A-431 (cells epidermis carcinoma), PC3 (a human prostate cancer) and T-47D (human breast cancer cell line). I hope that the research will encourage researcher to do more research to utilization of *Adansonia digitata*.

## Supplementary Information

Below is the link to the electronic supplementary material.

Supplementary Material 1

## Data Availability

Data are provided within the manuscript or supplementary information files.

## Abbreviations

HPLC: High Performance Liquid Chromatography
(MIC): Minimum inhibitory concentration
MBC: Minimum bactericidal concentration
Hela: cervical cancer
Hep G2: a human liver cancer
A549: lung cancer
A-431: cells epidermis carcinoma
PC3: a human prostate cancer
T-47D: human breast cancer
MTT: (3-(4,5-Dimethylthiazol-2-yl)-2,5-Diphenyltetrazolium Bromide) is used to assess cell viability
