# Supplementary material for: Reduce the risk of microbial activity and cytotoxicity by Adansonia digitata pulp extract grown under the semi arid conditions of Sudan
Source: Sci Rep. 2025 Dec 2;16:914. doi: 10.1038/s41598-025-30536-x (PMC12783721; doi:10.1038/s41598-025-30536-x)
Supplement: Supplementary file 1 — Supplementary Material 1 [file 41598_2025_30536_MOESM1_ESM.docx]

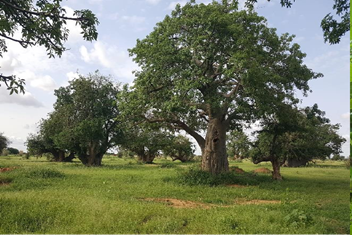

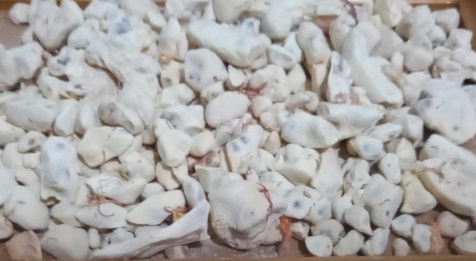
**Additional material**

Figure 1(a) .Adansonia digitata Tree (b).Adansonia digitata pulp


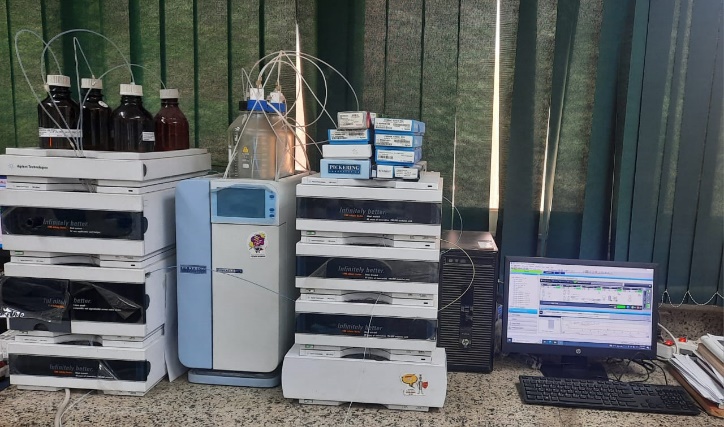


**Figure (2) High Performance Liquid Chromatography device (used in this study)**


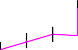

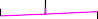


mAU

60

50

40

30

20

10

0

-10

2.5

5

7.5

10

12.5

15

17.5

20

22.5 min

3.572 - Gallic acid

4. 4.183 - Chlorogenic acid

423 - Catechin

5.413 - Methyl gallate 5.823 - Coffeic acid

6.313 - Syringic acid 6.722 - Rutin

7.102 - Ellagic acid

8.542 - Coumaric acid

8.967 - Vanillin

9.574 - Ferulic acid

10.194 - Naringenin

11.610 - Rosmarinic acid

19.112 - Cinnamic acid

20.502 - Kaempferol

21.095 - Hesperetin

Area: 248.328

**Figure (3) Phenolic compounds of *Adansonia* *digitata* pulp extract identified by HPLC.**


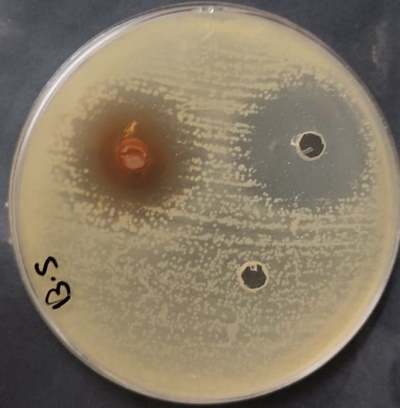


**Figure (4) Inhibitions zones (in mm) byAdansonia *digitata* ethanoic pulp extracts against *Bacillus* *subtilis*.**

**
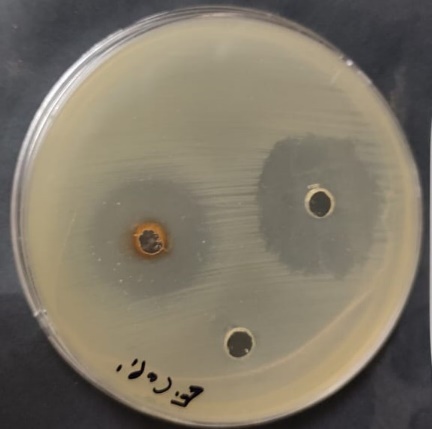
**

**Figure (5) Inhibitions zones (in mm) byAdansonia *digitata* ethanoic pulp extracts against *Escherichia* *coli*.**


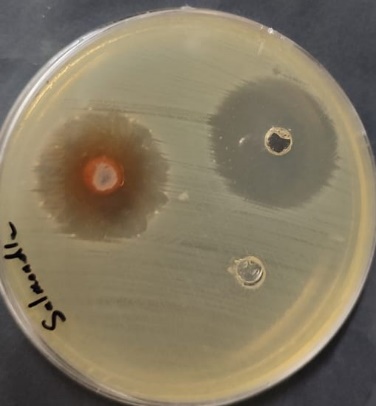

**Figure (6) Inhibitions zones (in mm) by *Adansonia* *digitata* ethanoic pulp extracts against *Salmonella* *typhi*.**


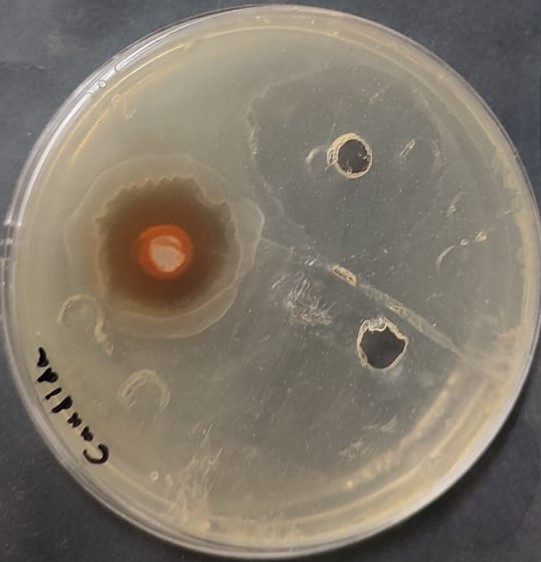


**Figure (7) Inhibitions zones (in mm) by *Adansonia* *digitata* ethanoic pulp extracts against fungi *Candida albicans.***


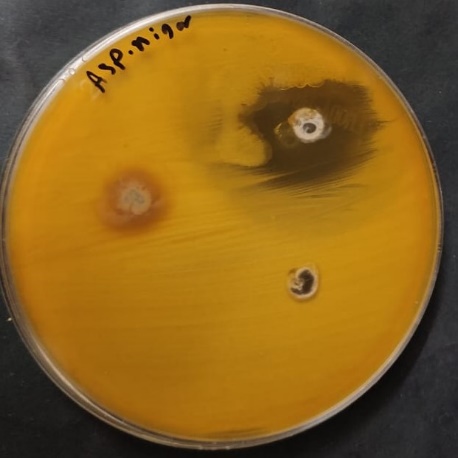


**Figure (8) Inhibitions zones (in mm) by *Adansonia* *digitata* ethanoic pulp extracts against fungi *Aspergillus* *niger***.


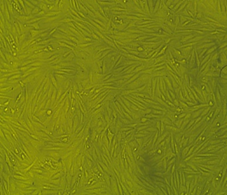


Hela cell Control

**Figure (9)** Effect of *Adansonia* *digitata* pulp extracts against Hela cell atdifferent concentration.


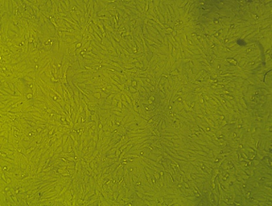


HepG2 cells Control

**Figure (10)** Effect of *Adansonia* *digitata* pulp extracts against HepG2 cell at different concentration.


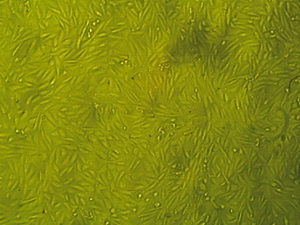


A549 cells control

**Figure (11)** Effect of *Adansonia* *digitata* pulp extracts against A549 cell at different concentration


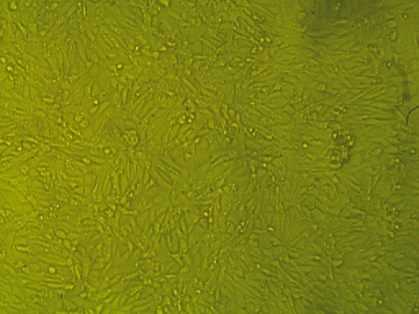


A-431cell control

**Figure (12)** Effect of *Adansonia* *digitata* pulp extracts against A431 cell at different concentration


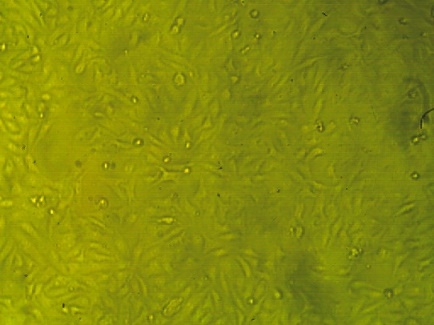


PC3 cell control

**Figure (13)** Effect of *Adansonia* *digitata* pulp extracts against Pc3 cell at different concentration


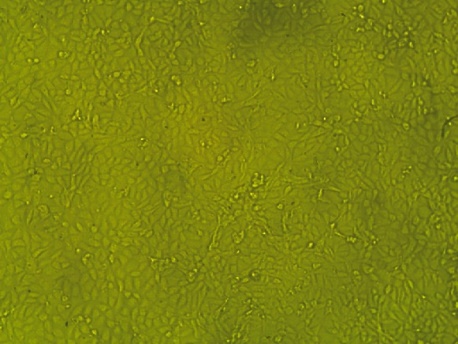


T47D cells control

**Figure (14)** Effect of *Adansonia* *digitata* pulp extracts against T47D cell at different concentration.
